# Trend of epidemiological indicators and factors associated with tuberculosis treatment dropout and death among street people in Brazil: an ecological and cross-sectional study, 2014-2022

**DOI:** 10.1590/S2237-96222024v34e20240273.en

**Published:** 2025-04-14

**Authors:** Jefferson Felipe Calazans Batista, Marcos Antonio Almeida-Santos, Sonia Oliveira Lima

**Affiliations:** 1 Universidade Tiradentes, Pós-graduação em Biociências e Saúde, Aracaju, SE, Brazil

**Keywords:** Tuberculosis, Street People, Epidemiological Indicators, Cross-Sectional Studies, Time Series Studies, Tuberculosis, Personas en Situación de Calle, Indicadores Epidemiológicos, Estudios Transversales, Estudios de Series Temporales

## Abstract

**Objective:**

To analyze the temporal trend of epidemiological indicators and factors associated with tuberculosis treatment dropout and death among street people.

**Methods:**

This is a cross-sectional study on tuberculosis among street people in Brazil from 2014-2022, using data from the Notifiable Health Conditions Information System (*Sistema de Informação de Agravos de Notificação*). We calculated cure, dropout, incidence and mortality indicators. The temporal trend of the indicators was estimated using Prais-Winsten regression, expressed via average percentage change (APC). Notification form variables were used to estimate factors associated with tuberculosis treatment dropout and death using logistic regression, resulting in odds ratios (OR) and 95% confidence intervals (95%CI).

**Results:**

In all, 21,904 tuberculosis cases were reported. In Brazil as a whole, APC for cure was -5.8% (95%CI -7.9; -3.8), while in the Southeast region, APC for incidence was -6.2% (95%CI -8.9; -3.5) and for mortality it was -5.3% (95%CI -10.0; -0.4). Predictors of dropout were: absence of directly observed treatment (OR 3.92; 95%CI 3.65; 4.20), being 20-39 years old (OR 1.81; 95%CI 1.41; 2.33) and use of illicit drugs (OR 1.57; 95%CI 1.46; 1.69). Factors associated with death were: absence of directly observed treatment (OR 3.18; 95%CI 2.86; 3.54), being >60 years old (OR 3.14; 95%CI 2.14; 4.61) and use of illicit drugs (OR 1.18; 95%CI 1.05; 1.34).

**Conclusion:**

The tuberculosis scenario in this population is worrying and its challenging context requires greater effort to achieve better screening and treatment, considering the multiple risk factors identified.

## Resumen

**Objetivo:**

Analizar la tendencia temporal de indicadores epidemiológicos y factores asociados al abandono del tratamiento y muerte por tuberculosis en personas en situación de calle.

**Métodos:**

Estudio transversal, sobre la tuberculosis en personas en situación de calle en Brasil en el período 2014-2022, utilizando datos del Sistema de Información de Enfermedades de Declaración Obligatoria. Se calcularon indicadores de curación y abandono, incidencia y mortalidad. La tendencia temporal de los indicadores se estimó mediante la regresión de Prais-Winsten, expresada mediante variación porcentual. Las variables presentes en el formulario de notificación se utilizaron para estimar los factores asociados con el abandono y la muerte por tuberculosis mediante regresión logística, lo que resulta en *odds ratios* (OR) e intervalos de confianza del 95% (IC 95%).

**Resultados:**

Se notificaron 21.904 casos de tuberculosis. La curación fue -5,8% (IC 95% -7,9; -3,8) en Brasil, la incidencia fue -6,2% (IC 95% -8,9; -3,5) y la mortalidad fue -5,3% (IC 95% -10,0; -0,4) en la región Sudeste. Los predictores de abandono fueron: ausencia de tratamiento directamente observado (OR 3,92; IC 95% 3,65; 4,20), edad de 20 a 39 años (OR 1,81; IC 95% 1,41; 2,33) y uso de drogas ilícitas (OR 1,57; IC 95% 1,46); 1,69). Los factores asociados a la muerte fueron: ausencia de tratamiento observado (OR 3,18; IC95% 2,86; 3,54), edad >60 años (OR 3,14; IC95% 2,14; 4,61) y consumo de drogas ilícitas (OR 1,18; IC95% 1,05; 1,34). ).

**Conclusión:**

El escenario de la tuberculosis en esta población es preocupante y su contexto desafiante requiere mayores esfuerzos para un mejor tamizaje y tratamiento, considerando los múltiples factores de riesgo identificados.

## Introduction

Tuberculosis is a global problem involving 10 million cases annually (1). In addition to immunological factors and viral load, the risk of becoming ill is linked to unfavorable living conditions. Therefore, the Ministry of Health classifies some populations as vulnerable, due to their greater risk of illness compared to the general population, such as Indigenous people, people deprived of liberty, people living with HIV/AIDS and people living on the streets (2).

Brazil is among the 30 countries with the highest levels of tuberculosis cases (1). Between 2006 and 2015, the average tuberculosis incidence rate in Brazil was 36.80 cases/100,000 inhabitants, with the Northern region having an incidence rate of 44.90/100,000, leading the list of Brazilian regions in terms of having the highest incidence rates, followed by the Southeast (39.80/100,000), Northeast (36.20/100,000), South (31.40/100,000) and Midwest (23.10/100,000) (3). Over time, tuberculosis incidence in Brazil reduced by 1.8% per year between 2001 and 2017 (4), mortality by 3.0% per year between 2005 and 2019 (5), while treatment dropout was stable between 2012 and 2018 (6), and percentage cure decreased by approximately 1.0% in the period 2001-2022 (7).

In 2022, there were around 236,000 street people registered on the Single Registry (Cadastro Único) for Social Programs, equivalent to 1.00 per 1,000 people in the Brazil (8). Street people are a priority for the Ministry of Health, as their risk of becoming ill from tuberculosis is 56 times higher when compared to the general population (2). The greatest risk of illness is due to factors such as social exclusion, extreme poverty, lack of perception about one’s own health, difficulties in accessing health services, stigma and prejudice, among others (9). 

In Brazil, in 2018, the tuberculosis cure rate was 34.5% among street people, while the treatment dropout rate was 32.6%, and the mortality rate was 10.0% (10), these being levels far removed from the targets established by the World Health Organization (WHO) (11). Therefore, it is relevant to assess factors that contribute to the unfavorable scenario of tuberculosis in this population, in addition to the temporal pattern of epidemiological indicators, aspects that can contribute to a better understanding of the phenomenon and review of public policies. There is a lack of studies on the subject (12), with most being focused on factors associated with tuberculosis outcomes among street people. As far as we know, in the literature, there are two Brazilian studies, one at state level (13) and the other at national level, conducted only with adults (14). As such, this research aimed to analyze the temporal trend of epidemiological indicators and factors associated with tuberculosis treatment dropout and death among street people.

## Methods

### Design and background

This is a cross-sectional study with data on tuberculosis in Brazil and its macro-regions from January 2014 to December 2022. In 2014, new variables were included on the Notifiable Health Conditions Diseases Information System (*Sistema de Informação de Agravos de Notificação* - SINAN), including data on street people.

### Participants

Confirmed cases of tuberculosis among street people were considered, according to data from the tuberculosis notification form. 

In order to extract cases of tuberculosis among street people, all cases of the disease were collected and those classified as “yes” in the “street person” variable field were selected. Therefore, the following cases were excluded, as they did not match the research objectives: (i) people who were not living on the streets; (ii) cases in which there was no information on the case closure status variable or that were classified as “regimen change”, “regimen failure”, “diagnosis change”, “drug-resistant tuberculosis” and “transfer”. Furthermore, two cases were excluded due to incorrect classification as per the International Classification of Diseases – 10th revision (ICD-10), namely, A24 and A31, as well as two duplicate cases (Supplementary Figure 1).

### Variables

The dependent variable was treatment dropout, resulting from grouping together treatment dropout and primary treatment dropout.

The following variables were included: age group (in years: <19, 20-39, 40-59, ≥60), sex (male, female, unknown), region of the country (North, Northeast, Southeast, South and Midwest), year of notification (2014-2022), race/skin color (White, Black, mixed race, Asian and Indigenous, unknown), schooling (illiterate, incomplete elementary education, complete elementary education, incomplete high school education, complete high school education, incomplete higher education and complete higher education, unknown); clinical variables – type of entry (new case, retreatment), clinical form (pulmonary, extrapulmonary, mixed, unknown), AIDS (yes, no, unknown), diabetes (yes, no, unknown), illicit drugs (yes, no, unknown ), tobacco smoking (yes, no, unknown), directly observed treatment (yes, no, unknown), sputum smear microscopy (positive, negative, not performed, inconclusive, not applicable), sputum culture (positive, negative, not performed, inconclusive, not applicable), HIV serology (positive, negative, not performed, inconclusive, not applicable), rapid molecular testing (positive for rifampicin-sensitive tuberculosis; rifampicin-resistant; not performed; negative, unknown), case closure (cure, treatment dropout, primary treatment dropout and death due to tuberculosis).

Additionally, an empirical classification system was calculated to evaluate the quality of case follow-up in accordance with the recommendations of the Brazilian Society of Pulmonology and Phthisiology (*Sociedade Brasileira de Pneumologia e Tisiologia*) (15). The indicator combines five recommendations: (1) whether the reported case underwent control sputum smear microscopy in the 2nd month of treatment; (2) control sputum smear microscopy in the 4th month of treatment; (3) control sputum smear microscopy in the 6th month of treatment; (4) whether there was a record of contact examinations; and (5) whether directly observed treatment took place. Classification of follow-up quality is divided into four, namely: insufficient, regular, good and excellent. The dependent variable was case closure (cure, treatment dropout and death due to tuberculosis).

In the aggregated data, the following epidemiological indicators were calculated, according to the Ministry of Health standard (2): incidence rate and mortality rate, percentage cure and percentage treatment dropout. Incidence and mortality rates were calculated using the following formula:


$$\frac{o_{i}}{p_{i}}\times 100,000$$

Where: o_i_ – new tuberculosis cases or deaths among street people in a given place and period; and p_i_ – street people in the same place and period. For the purposes of this calculation alone, only cases classified as new case, post-death and unknown were considered, in the “type of entry” variable. Furthermore, deaths were not extracted from the Mortality Information System (*Sistema de Informação sobre Mortalidade* - SIM), due to the impossibility of classifying deaths according to street people because this variable field does not exist on the System. 

Cure and dropout percentages were calculated using the following formula:


$$\frac{o_{i}}{t_{i}}\times 100$$

Where: o_i_ – crude occurrences of total cases that progressed to cure and treatment dropout among street people, in a given place and period; and t_i_ – total tuberculosis cases among street people in the same place and period.

### Data source and measurement

Data relating to confirmed tuberculosis cases were extracted from the SINAN system, while the estimate of the number of street people came from the Institute of Applied Economic Research (*Instituto de Pesquisa Econômica Aplicada*), estimated through collection of official data from municipal governments, number of street people registered on the Single Registry and projections based on previous data. This information is available for the period from 2012 to 2022, for Brazil as a whole, its macro-regions and municipalities (municipality size ranging from small I to metropolis) (16).

All data was accessed in September 2023, as follows (Supplementary Figure 1):

- Access to the annual databases (2014 to 2022) of tuberculosis notification forms on the website of the Brazilian National Health System Information Technology Department – data in .dbc format;
- Import of downloaded data for conversion to .dbf format, using TabWin;
- Merger of the annual databases (2014-2022) using TabWin, in its “View .dbf” -> “Add records” section;
- Data tabulation by year and sociodemographic characteristics (aggregated data);
- Microdata exportation for treatment on a Microsoft Excel spreadsheet.

With regard to the HIV serology, sputum culture and sputum smear microscopy variables, the inconclusive and not applicable classifications, in addition to data classified as unknown in the other variables, were considered to be data with no information (missing).

### Bias

Considering the presence of missing data in the study and with the aim of improving the predictive model, a multiple imputation procedure was carried out, assuming that losses were random, this being a common fact in studies of this nature (17). By including all available variables and using the predictive matching method, ten imputation models were proposed for greater power and precision (18).

### Data analysis

Temporal trend was estimated using Prais-Winsten regression. All epidemiological indicators were transformed into a base 10 logarithmic scale. Based on the results, annual percentage change (APC) and 95% confidence intervals (95%CI) were estimated (19).

Percentage change was interpreted as follows (19):

- Rising trend: positive change and statistically significant model (p-value<0.05);
- Falling trend: negative variation and statistically significant model (p-value<0.05);
- Stationary trend: model not significant (p-value>0.05).

In this research, following autocorrelation correction, Durbin-Watson values were reported to ensure trend interpretations, considering values between 1.5 and 2.5 as reliable. The trends were calculated using Stata version 17.

Estimation of associated factors was performed by means of multinomial logistic regression, using the enter input method. The closure status of cases classified as cure, treatment dropout and death due to tuberculosis was taken as a dependent variable.

The “cure” category was used as a reference, being compared with the treatment dropout and death due to tuberculosis categories. 

Initially, a logistic regression model was proposed with all variables included to calculate the odds ratios (OR) and 95%CI, and multiple models were progressively tested, by removing and reincluding variables, aiming to adjust for the model’s potential for confounders and interaction. In order to compare the tests, we analyzed the Bayesian information criterion and Nagelkerke R2 (pseudo R-squared). The mean of the Bayesian criterion of the ten imputed databases was considered as a comparative measure. The adjusted model included all predictors from the final model as adjustment variables.

Crude and adjusted odds ratios (OR) were calculated. The analyses were performed using the Statistical Package for Social Science version 25.

## Results

The study included 21,904 street people with tuberculosis, which represents 2.7% of total tuberculosis cases reported in Brazil as a whole (n=820,247). The Southeast region had the highest proportion of notifications, followed by the South, Northeast, North and Midwest regions (Table 1). Furthermore, missing data accounted for 6.3% (Supplementary Table 1).

**Table 1:** Number of tuberculosis cases among street people, by sociodemographic and clinical variables. Brazil, 2014-2022 (n=21,904)

Characteristics	n (%)
**Age group** (years)	
<19	348 (1.6)
>60	1,258 (5.7)
20-39	10,928 (49.9)
40-59	9,370 (42.8)
Sex	
Female	3,996 (18.2)
Male	17,907 (81.8)
No information	1 (0.0)
**Race/skin color**	
Asian/Indigenous	233 (1.1)
White	5,512 (25.2)
Black	14,299 (65.3)
No information	1,860 (8.5)
Schooling	
Illiterate	978 (4.5)
Elementary	9,745 (44.5)
High school	2,770 (12.6)
No information	8,022 (36.6)
Higher education	389 (1.8)
**Region**	
Midwest	884 (4.0)
Northeast	3,315 (15.1)
North	1,124 (5.1)
Southeast	12,814 (58.5)
South	3,767 (17.2)
**Type of entry**	
New case	8,874 (40.5)
Retreatment	12,736 (58.1)
No information	294 (1.3)
**Clinical form**	
Extrapulmonary	741 (3.4)
Mixed	658 (3.0)
Pulmonary	20,503 (93.6)
No information	2 (0.0)
Aids	
No	16,154 (73.7)
No information	1,515 (6.9)
Yes	4,235 (19.3)
Alcoholism	
No	9,083 (41.5)
No information	1,036 (4.7)
Yes	11,785 (53.8)
Diabetes	
No	19,699 (89.9)
No information	1,443 (6.6)
Yes	762 (3.5)
**Illicit drugs**	
No	8,188 (37.4)
No information	1,198 (5.5)
Yes	12,518 (57.1)
**Tobacco smoking**	
No	10,676 (48.7)
No information	1,420 (6.5)
Yes	9,808 (44.8)
**Directly observed treatment**	
No	8,756 (40.0)
No information	5,903 (26.9)
Yes	7,245 (33.1)
**Sputum smear microscopy**	
Not performed	3,839 (17.5)
Negative	5,681 (25.9)
Positive	11,954 (54.6)
No information	430 (2.0)
**Sputum culture**	
Not performed	2,174 (9.9)
Negative	10,681 (48.8)
Positive	8,456 (38.6)
No information	593 (2.7)
**HIV serology**	
Not performed	13,863 (63.3)
Negative	3,243 (14.8)
Positive	4,543 (20.7)
No information	255 (1.2)
**Follow-up**	
Good	244 (1.1)
Excellent	32 (0.1)
Insufficient	18,955 (86.5)
Regular	2,673 (12.2)
**Rapid molecular testing**	
Not performed	9,799 (44.7)
Negative	1,111 (5.1)
Positive for rifampicin-sensitive tuberculosis	8,606 (39.3)
Rifampicin-resistant	179 (0.8)
No information	2,209 (10.1)
**Case closure**	
Treatment dropout	11,260 (51.4)
Cure	8,653 (39.5)
Death due to tuberculosis	1,991 (9.1)

**Figure 1:**
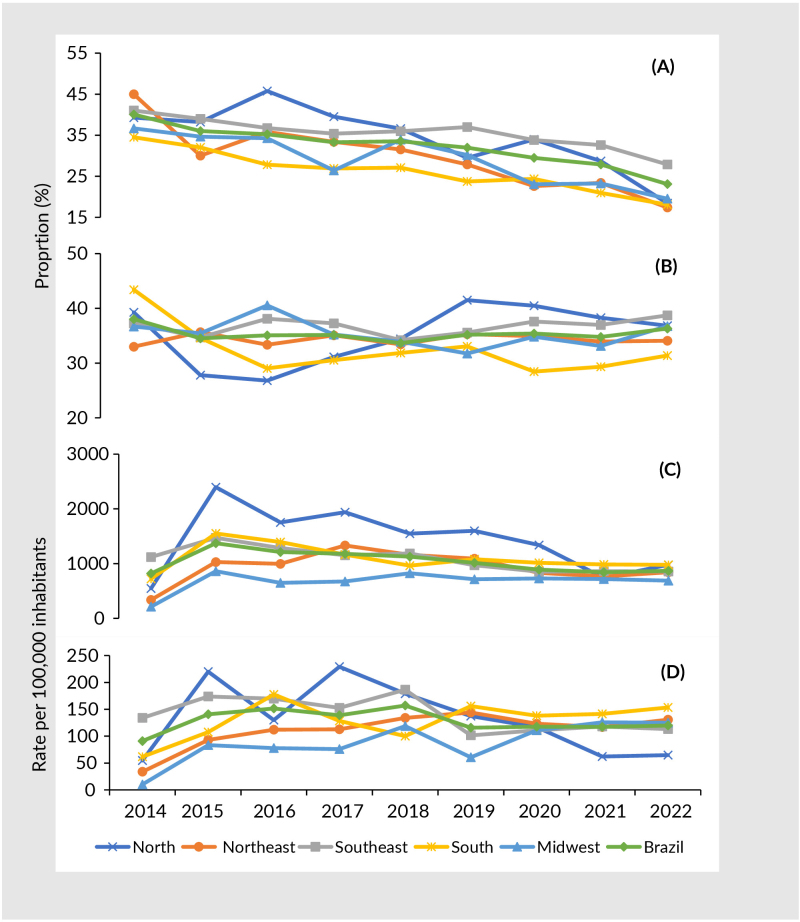
Cure (A) and treatment dropout (B) percentages, incidence (C) and mortality (D) rates per 100,000 inhabitants for tuberculosis among street people, by macro-region. Brazil, 2014-2022 (n=21,904)

In Brazil as a whole, the cure rate was 31.3%; treatment dropout was 35.2%; the incidence rate was 1,007.81 cases per 100,000 street people; and the tuberculosis mortality rate was 126.99/100,000. In the Southeast region, there was a considerable reduction in the incidence rate from 2019 onwards (Supplementary Table 2). A pattern of falling trends in percentage cure and incidence rates was seen, while treatment dropout and mortality appeared to stagnate (Figure 1).

The majority of the street people studied were males (81.8%), young adults (49.9%), Black (65.3%), with elementary education (44.5%) and living in the Southeast region (58.5%). The predominant clinical characteristics were: cases undergoing retreatment (58.1%), pulmonary clinical form (93.6%), alcoholism (53.8%), tobacco smoking (48.7%) and illicit drug use (57 .1%), absence of directly observed treatment (40.0%), positive sputum smear microscopy (54.6%), negative sputum culture (48.8%), HIV serology not performed (63.3%), and insufficient follow-up (86.5%) (Table 1).

Percentage cure showed a falling trend throughout Brazil as a whole (APC -5.8; 95%CI -7.9; -3.8), in particular in the Northeast region (APC -8.5; 95%CI -11.0; -6.0) and the Northern region (APC -7.7; 95%CI -12.2; -2.8). The treatment dropout indicator showed a stationary trend, while in the Southeast region incidence (APC -6.2; 95%CI -8.9; -3.5) and mortality decreased (APC -5.3; 95% CI -10.0; -0.4). There was no evidence of autocorrelation biases in these models (Durbin-Watson: >1.5) (Table 2). 

**Table 2:** Annual percentage change (APC) and confidence intervals (95%CI) of the cure and treatment dropout percentages and incidence and mortality rates (per 100,000) for tuberculosis among street people. Brazil, 2014-2022 (n=21,904)

Indicators	APC (95%CI)	p-value	Durbin-Watson	Interpretation
**Percentage cure**				
North	-7.7 (-12.2; -2.8)	0.008	1.465	Falling
Northeast	-8.5 (-11.0; -6.0)	<0.001	1.762	Falling
Southeast	-4.0 (-5.9; -1.9)	0.003	1.403	Falling
South	-6.8 (-8.2; -5.3)	<0.001	1.801	Falling
Midwest	-6.9 (-9.5; -4.3)	<0.001	2.020	Falling
Brazil	-5.8 (-7.9; -3.8)	<0.001	1.421	Falling
**Percentage dropout**				
North	2.4 (-2.9; 7.9)	0.323	1.525	Stationary
Northeast	0.1 (-0.4; 0.4)	0.818	1.765	Stationary
Southeast	0.4 (-0.7; 1.6)	0.388	1.921	Stationary
South	-3.0 (-6.5; 0.6)	0.087	1.382	Stationary
Midwest	-1.1 (-2.9; 0.8)	0.209	1.804	Stationary
Brazil	-0.2 (-1.1; 0.7)	0.623	1.351	Stationary
**Incidence rate**				
North	-3.9 (-16.6; 10.9)	0.535	1.379	Stationary
Northeast	4.9 (-8.9; 20.9)	0.449	1.523	Stationary
Southeast	-6.2 (-8.9; -3.5)	<0.001	1.554	Falling
South	-2.0 (-7.9; 4.7)	0.454	1.504	Stationary
Midwest	6.4 (-3.3; 17.1)	0.169	1.273	Stationary
Brazil	-3.5 (-8.2; 1.3)	0.128	1.369	Stationary
**Mortality rate**				
North	-6.2 (-20.7; 11.1)	0.401	1.466	Stationary
Northeast	12.6 (-0.3; 27.1)	0.053	1.435	Stationary
Southeast	-5.3 (-10.0; -0.4)	0.038	1.816	Falling
South	7.2 (-1.4; 16.5)	0.091	1.645	Stationary
Midwest	19.7 (2.5; 40.0)	0.029	1.397	Rising

Four multinomial logistic regression models were proposed, with the AIDS and macro-region follow-up indicator variables having been removed (Supplementary Table 3). The final model was statistically significant (p-value<0.001) (Tables 3 and 4).

**Table 3:** Crude and adjusted odds ratios (OR) and 95% confidence intervals (95%CI) for tuberculosis treatment dropout, by sociodemographic and clinical variables, among street people. Brazil, 2014-2022 (n=21,904)

Variable	Crude OR (95%CI)	p-value	Adjusted OR (95%CI)	p-value
**Age group** (years)				
<19	1.00		1.00	
>60	0.69 (0.54; 0.89)	0.005	0.79 (0.59; 1.05)	0.107
20-39	2.10 (1.68; 2.62)	<0.001	1.69 (1.31; 2.17)	<0.001
40-59	1.30 (1.04; 1.63)	0.022	1.18 (0.92; 1.53)	0.191
Sex				
Female	1.00		1.00	
Male	0.76 (0.71; 0.82)	<0.001	1.00 (0.92; 1.09)	0.966
**Race/skin color**				
Asian/Indigenous	0.68 (0.51; 0.90)	0.007	0.71 (0.51; 1.00)	0.051
White	1.00		1.00	
Black	1.11 (1.04; 1.19)	0.002	1.07 (0.99; 1.16)	0.089
Schooling				
Illiterate	1.25 (0.96; 1.62)	0.099	1.43 (1.08; 1.88)	0.015
Elementary	1.29 (1.02; 1.64)	0.038	1.21 (0.93; 1.56)	0.156
High school	1.13 (0.87; 1.46)	0.361	1.01 (0.77; 1.31)	0.958
Higher education	1.00		1.00	
**Type of entry**				
New case	1.00		1.00	
Retreatment	0.51 (0.48; 0.54)	<0.001	0.55 (0.51; 0.58)	<0.001
**Clinical form**				
Extrapulmonary	0.74 (0.63; 0.86)	<0.001	0.65 (0.54; 0.77)	<0.001
Mixed	1.09 (0.92; 1.30)	0.312	0.86 (0.71; 1.05)	0.138
Pulmonary	1.00		1.00	
Alcoholism				
No	1.00		1.00	
Yes	1.04 (0.98; 1.10)	0.23	0.95 (0.88; 1.02)	0.166
Diabetes				
No	1.00		1.00	
Yes	0.74 (0.63; 0.86)	<0.001	0.87 (0.74; 1.03)	0.109
**Illicit drugs**				
No	1.00		1.00	
Yes	1.83 (1.73; 1.95)	<0.001	1.57 (1.46; 1.69)	<0.001
**Tobacco smoking**				
No	1.00		1.00	
Yes	1.20 (1.13; 1.27)	<0.001	1.11 (1.03; 1.20)	0.009
**Directly observed treatment**				
No	4.05 (3.80; 4.32)	<0.001	3.93 (3.67; 4.20)	<0.001
Yes	1.00		1.00	
**Sputum smear microscopy**				
Not performed	0.84 (0.77; 0.91)	<0.001	0.93 (0.84; 1.02)	0.135
Negative	1.00		1.00	
Positive	0.83 (0.77; 0.88)	<0.001	0.90 (0.83; 0.98)	0.01
**Sputum culture**				
Not performed	0.58 (0.53; 0.64)	<0.001	0.55 (0.49; 0.62)	<0.001
Negative	1.00		1.00	
Positive	0.69 (0.65; 0.73)	<0.001	0.68 (0.63; 0.73)	<0.001
**HIV serology**				
Not performed	0.40 (0.36; 0.44)	<0.001	0.42 (0.38; 0.47)	<0.001
Negative	1.00		1.00	
Positive	0.79 (0.71; 0.88)	<0.001	0.64 (0.57; 0.72)	<0.001
**Rapid molecular testing**				
Not performed	0.98 (0.86; 1.11)	0.727	0.88 (0.76; 1.01)	0.074
Negative	1.00		1.00	
Rifampicin-resistant	1.12 (0.80; 1.58)	0.501	0.98 (0.67; 1.43)	0.902
Rifampicin-sensitive	1.02 (0.90; 1.16)	0.701	1.11 (0.96; 1.28)	0.173

Greater odds of treatment dropout among street people were found in individuals aged 20-39 years (OR 0.69; 95%CI 1.31; 2.17), who used illicit drugs (OR 1.57; 95 %CI 1.46; 1.69) and did not have directly observed treatment (OR 3.93; 95%CI 3.67; 4.20) (Table 3). Greater odds of death were identified among people over 60 years old (OR 3.10 95%CI 1.96; 4.90), infected with the mixed clinical form mixed (OR 2.66; 95%CI 2.05; 3 .46), who used alcohol (OR 1.34; 95%CI 1.18; 1.51), who used illicit drugs (OR 1.18; 95%CI 1.05; 1.34) and did not have directly observed treatment (OR 3.46; 95%CI 2.98; 4.02) (Table 4).

**Table 4:** Crude and adjusted odds ratios (OR) and 95% confidence intervals (95%CI) for tuberculosis deaths, by sociodemographic and clinical variables, among street people. Brazil, 2014-2022 (n=21.904)

Variable	Crude OR (95%CI)	p-value	Adjusted OR (95%CI)	p-value
**Age group** (years)				
<19	1.00		1.00	
>60	2.91 (1.89; 4.46)	<0.001	3.10 (1.96; 4.90)	<0.001
20-39	1.15 (0.76; 1.74)	0.498	1.29 (0.84; 2.00)	0.249
40-59	1.65 (1.09; 2.48)	0.017	1.82 (1.18; 2.81)	0.007
Sex				
Female	1.00		1.00	
Male	1.37 (1.19; 1.58)	<0.001	1.25 (1.07; 1.46)	0.005
**Race/skin color**				
Asian/Indigenous	0.89 (0.56; 1.42)	0.636	0.79 (0.48; 1.29)	0.34
White	1.00		1.00	
Black	1.06 (0.94; 1.19)	0.336	1.03 (0.91; 1.16)	0.696
Schooling				
Illiterate	4.59 (2.36; 8.92)	<0.001	4.13 (2.11; 8.08)	<0.001
Elementary	2.99 (1.65; 5.43)	<0.001	2.88 (1.62; 5.13)	<0.001
High school	2.38 (1.32; 4.29)	0.006	2.41 (1.37; 4.24)	0.004
Higher education	1.00		1.00	
**Type of entry**				
New case	1.00		1.00	
Retreatment	0.88 (0.79; 0.98)	0.019	0.78 (0.69; 0.87)	<0.001
**Clinical form**				
Extrapulmonary	1.05 (0.82; 1.35)	0.688	0.96 (0.73; 1.28)	0.793
Mixed	2.14 (1.90; 2.42)	<0.001	2.66 (2.05; 3.46)	<0.001
Pulmonary	1.00		1.00	
Alcoholism				
No	1.00		1.00	
Yes	1.27 (1.14; 1.41)	<0.001	1.34 (1.18; 1.51)	<0.001
Diabetes				
No	1.00		1.00	
Yes	1.28 (1.01; 1.62)	0.044	1.07 (0.83; 1.37)	0.611
**Illicit drugs**				
No	1.00		1.00	
Yes	0.89 (0.81; 0.99)	0.029	1.18 (1.05; 1.34)	0.008
**Tobacco smoking**				
No	1.00		1.00	
Yes	0.97 (0.88; 1.08)	0.583	0.91 (0.81; 1.03)	0.139
**Directly observed treatment**				
No	3.46 (2.95; 4.06)	<0.001	3.46 (2.98; 4.02)	<0.001
Yes	1.00		1.00	
**Sputum smear microscopy**				
Not performed	0.44 (0.37; 0.51)	<0.001	0.52 (0.44; 0.62)	<0.001
Negative	1.00		1.00	
Positive	0.63 (0.57; 0.70)	<0.001	0.70 (0.62; 0.79)	<0.001
**Sputum culture**				
Not performed	0.27 (0.22; 0.33)	<0.001	0.35 (0.28; 0.44)	<0.001
Negative	1.00		1.00	
Positive	0.38 (0.34; 0.42)	<0.001	0.46 (0.41; 0.52)	<0.001
**HIV serology**				
Not performed	0.25 (0.22; 0.29)	<0.001	0.32 (0.28; 0.37)	<0.001
Negative	1.00		1.00	
Positive	0.15 (0.12; 0.18)	<0.001	0.16 (0.13; 0.19)	<0.001
**Rapid molecular testing**				
Not performed	2.66 (1.94; 3.63)	<0.001	1.75 (1.26; 2.43)	<0.001
Negative	1.00		1.00	
Rifampicin-resistant	1.83 (0.96; 3.52)	0.069	1.43 (0.71; 2.87)	0.318
Rifampicin-sensitive	1.60 (1.18; 2.18)	0.003	1.44 (1.05; 1.99)	0.025

## Discussion

This research identified a worrying and unfavorable scenario of tuberculosis among street people, with epidemiological indicators far removed from the targets established by the WHO, namely: at least 85% cure, maximum dropout of 5% and 1.00 death per 100,000 inhabitants (11). An annual reduction in cure rates was identified across the Brazil as a whole, while a reduction in incidence and mortality occurred only in the Southeast region. Factors that most increase the odds of dropout are absence of directly observed treatment, being in the 20-39 age group and use of illicit drugs, while the odds of death are higher among people over 60 years of age, illiterate people, those with the mixed clinical form and without directly observed treatment.

The main limitation of this study refers to the use of secondary data, due to underreporting and incomplete data, especially in this population, which can be difficult to reach and follow-up on. Data regarding deaths would be more reliable if it could be obtained from the Mortality Information System. However, with that system it is not possible to identify street people. Another limitation refers to the possible lower quality of the data in 2014, when the new variables were included, in addition to the period 2020-2022, when there was an increase in underreporting due to the COVID-19 pandemic.

The incidence and mortality rates, as well as the percentage cure and treatment dropout rates among street people identified in this research are unfavorable and discrepant, compared to other vulnerable groups. In Brazil, incidence among people deprived of liberty was 540.90/100,000 in the period 2013-2015 (20), while in a survey on Indigenous people (2011-2017), average incidence was 109.00/100,000 (21). 

The incidence rate we found was also higher than the rate of 36.00/100,000 identified in the United States (2011-2016) (22). A possible explanation for this discrepancy is the combination of underreporting and greater social vulnerability in Brazil. Factors such as greater population density in urban areas, less effective public policies and barriers to accessing health services can contribute to higher incidence rates. Tuberculosis in this population is complex, when compared to the rest of the population, due to the existence of unsafe situations, inadequate rest and food, presence of HIV/AIDS, high blood pressure and drug use, which are recurrent in this group and contribute to difficulties in treatment, increasing the likelihood of unfavorable outcomes (9). This reality is made worse by marginalization, inadequate reception by public services, detachment from public policies and difficult access to health services, this being a fact that compromises health care and leads to negative contexts.

In this research, percentage cure showed a reduction in the last nine years in both Brazil as a whole and it the country’s macro-regions. The literature lacks evidence on the trend of cured tuberculosis among street people; however, among people living with HIV, the trend pattern was similar, with a trend falling by 1.1% (95%CI -1.4; -0.8) per year between 2002-2021 in Brazil as a whole (23). The reduction in the cure rate is worrying and suggests the need for immediate interventions by public health authorities. This scenario may reflect ongoing difficulties in improving treatment adherence, especially among street people, which are made worse by social and economic issues that directly impact individuals’ ability to follow strict therapeutic regimens. Therefore, treating the disease in this population demands more intense care and support on the part of health professionals and institutions. Lack of adherence is a serious problem and is seen as being multi-causal (24); therefore, strengthening tuberculosis control strategies should be focused on prevention, screening, active tracing of cases and improving follow-up until cure is achieved.

Incidence and mortality rates showed annual reduction in the Southeast region. Similar results were found in a study on incidence in the Southeastern states between 2001 and 2017 (4) and on mortality from 2005 to 2019 (5). This can be explained by the greater effectiveness of public policies in the region, in addition to the strengthening of tuberculosis control programs, combined with investments in health infrastructure and greater primary care coverage. Notwithstanding, the Southeast region had higher concentrations of street people who are exposed to precarious living conditions and comorbidities (25). Social protection policies for people with tuberculosis is relevant for strengthening support for street people through social inclusion, guaranteeing access to social and human rights and combating poverty (26). The National Policy for the Population Living on the Streets (*Política Nacional para a População em Situação de Rua*), in turn, aims to promote respect for human dignity, integration of public policies at all levels of government, and broad and safe access to health services, social support and income transfer programs (27). Despite this scenario, the challenges for controlling tuberculosis in this population are still diverse, such as diminished human resources, professional unpreparedness, and care fragmentation. 

Absence of directly observed treatment was a predictor of increased odds of tuberculosis treatment dropout and death, and was provided to less than half of the population. A similar result was found in the United States between 2014 and 2016, where the odds of completing latent tuberculosis treatment with directly observed treatment were 40% higher when compared to treatment self-administration among street people (28). The WHO highlights this strategy as important for increasing favorable outcomes (29). It is a municipal and local government role to identify the best strategies for approaching this group, with intersectorality being a basic requirement for improved indicators. Mapping care networks, seeking partnerships for detection, diagnosis and treatment, defining reference services, active tracing and training the professionals involved are relevant for mitigating negative tuberculosis outcomes (30). Provision of aid to reduce the economic impact of treatment or guarantee transport and food is important for ensuring comprehensive and long-term care.

In this study, we found that more than half of the population used alcohol, tobacco and illicit drugs, and these factors were related to an increase in unfavorable tuberculosis outcomes. These results corroborate a study carried out in São Paulo (13). Use of such substances contributes to increasing this population’s vulnerability to tuberculosis and, consequently, abuse of these substances is heightened by social determinants, such as the way of living on the streets, the struggle for survival, poor sleeping conditions, poverty and stigma (12). Although the Brazilian health system is public, it must be considered that this population places priority on its quest for basic living conditions, such as shelter and food (9), which can delay and make it difficult to seek health care. Of relevance in these cases are strategies such as integrating social programs intended help people with substance use disorder with health services, through educational campaigns, harm reduction, rehabilitation and social reintegration.

The mixed clinical form of tuberculosis doubled the odds of death, compared to the pulmonary form. Similar data were found in a Brazilian study (n=2,096) conducted with a young Indigenous population between 2006 and 2016 (OR 5.60; 95%CI 2.80; 11.40) (31). The extrapulmonary form is particularly more difficult to identify, due to the low pulmonary bacterial load and the challenge in collecting sputum samples (32), in addition to the presence of atypical signs and symptoms. Cases of mixed tuberculosis deserve attention, considering that it is common among immunocompromised people living with HIV/AIDS (33). Considering the complexity of screening for the extrapulmonary form, improvements in health care networks are suggested for active case tracing and individualized treatment suited to the needs of street people, with the aim of reducing likelihood of death. 

Percentage tuberculosis cure showed a reduction throughout Brazil as a whole, in particular in the Northeast and North regions, between 2014 and 2022. The dropout indicator did not show annual change, while incidence and mortality only showed a falling trend in the Southeast region. Furthermore, there was a rising annual mortality trend in the Midwest region. Several factors contributed to greater risk of tuberculosis treatment dropout and death, namely: age group, illiteracy, case retreatment, use of illicit drugs, tobacco smoking, alcoholism and absence of directly observed treatment. 
