# Supplementary material for: Trend of epidemiological indicators and factors associated with tuberculosis treatment dropout and death among street people in Brazil: an ecological and cross-sectional study, 2014-2022
Source: Epidemiol Serv Saude. 2025 Apr 14;34:e20240273. doi: 10.1590/S2237-96222024v34e20240273.en (PMC11998906; doi:10.1590/S2237-96222024v34e20240273.en)
Supplement: Translation

# Translation: Tendência dos indicadores epidemiológicos e fatores associados ao abandono de tratamento e óbito pela tuberculose em pessoas em situação de rua no Brasil: estudo ecológico e transversal, 2014-2022

**DOI:** 10.1590/S2237-96222024v34e20240273.pt

**Authors:** Jefferson Felipe Calazans Batista, Marcos Antonio Almeida-Santos, Sonia Oliveira Lima

**Affiliations:** 1 Universidade Tiradentes, Pós-graduação em Biociências e Saúde, Aracaju, SE, Brasil

**Keywords:** Tuberculose, Pessoas em Situação de Rua, Indicadores Epidemiológicos, Estudos Transversais, Estudos de Séries Temporais

## Abstract

**Objetivo:**

Analisar a tendência temporal dos indicadores epidemiológicos e os fatores associados ao abandono de tratamento e óbito pela tuberculose em pessoas em situação de rua.

**Métodos:**

Estudo, transversal sobre tuberculose em pessoas em situação de rua no Brasil de 2014-2022, usando dados do Sistema de Informação de Agravos de Notificação. Calcularam-se os indicadores de cura e abandono, incidência e mortalidade. Estimou-se a tendência temporal dos indicadores pela regressão de Prais-Winsten, expressa via variação percentual. Utilizaram-se variáveis presentes na ficha de notificação para estimar os fatores associados ao abandono e ao óbito pela tuberculose usando regressão logística, que resulta na razão de chances (*odds ratio*, OR) e intervalo de confiança de 95% (IC95%).

**Resultados:**

Foram notificados 21.904 casos de tuberculose. A cura foi -5,8% (IC95% -7,9; -3,8) no Brasil, a incidência, -6,2% (IC95% -8,9; -3,5) e a mortalidade, -5,3% (IC95% -10,0; -0,4) na região Sudeste. Os preditores do abandono foram: tratamento diretamente observado ausente (OR 3,92; IC95% 3,65; 4,20), 20-39 anos (OR 1,81; IC95% 1,41; 2,33) e uso de drogas ilícitas (OR 1,57; IC95% 1,46; 1,69). Os fatores associados ao óbito foram: tratamento observado ausente (OR 3,18; IC95% 2,86; 3,54), >60 anos (OR 3,14; IC95% 2,14; 4,61) e uso de drogas ilícitas (OR 1,18; IC95% 1,05; 1,34).

**Conclusão:**

O cenário da tuberculose nessa população é preocupante e seu contexto desafiador requer mais esforços para melhor rastreamento e tratamento, considerando-se os múltiplos fatores de risco identificados.

## Introdução

A tuberculose é um problema mundial responsável por 10 milhões de casos anualmente (1). Além de fatores imunológicos e da carga viral, o risco de adoecer está ligado a condições de vida desfavoráveis. Portanto, o Ministério da Saúde classifica algumas populações como vulneráveis, devido a seu maior risco de adoecimento em comparação com a população geral, como indígenas, pessoas privadas de liberdade, pessoas que vivem com HIV/aids e pessoas em situação de rua (2).

O Brasil está entre os 30 países com maiores casos de tuberculose (1). No período de 2006 a 2015, a taxa média de incidência de tuberculose no Brasil foi de 36,80 casos/100 mil habitantes, tendo a região Norte, incidência de 44,90/100 mil, liderando a lista das regiões com maior ocorrência, seguida pelas regiões Sudeste (39,80/100 mil), Nordeste (36,20/100 mil), Sul (31,40/100 mil) e Centro-Oeste (23,10/100 mil) (3). Temporalmente, a incidência da tuberculose no Brasil se reduziu 1,8% ao ano entre 2001 e 2017 (4), a mortalidade, 3,0% ao ano de 2005-2019 (5), enquanto o abandono de tratamento se mostrou estável entre 2012 e 2018 (6), e as proporções de cura diminuíram aproximadamente 1,0% no período de 2001-2022 (7).

No ano de 2022, havia cerca de 236 mil pessoas em situação de rua, inscritas no Cadastro Único para programas sociais, o equivalente a 1,00 em cada 1 mil pessoas no país (8). Pessoas em situação de rua são prioridade para o Ministério da Saúde, por apresentarem um risco de adoecer por tuberculose 56 vezes maior em comparação à população geral (2). O maior risco de adoecimento é decorrente de fatores como exclusão social, pobreza extrema, falta de percepção sobre a própria saúde, dificuldades no acesso aos serviços de saúde, estigma e preconceito, entre outros (9). 

No Brasil, em 2018, o percentual de cura da tuberculose foi de 34,5% nas pessoas em situação de rua, enquanto o de abandono de tratamento foi de 32,6%, e o de óbito, de 10,0% (10), níveis longe das metas estabelecidas pela Organização Mundial da Saúde (OMS) (11). Portanto, torna-se relevante a avaliação dos fatores que contribuem para o cenário desfavorável da doença nessa população, além do padrão temporal dos indicadores epidemiológicos, aspectos que podem contribuir para melhor compreensão do fenômeno e revisão de políticas públicas. Há escassez de estudos sobre a temática (12), principalmente voltados aos fatores associados aos desfechos da tuberculose em pessoas em situação de rua. Até onde se sabe, na literatura, há dois estudos brasileiros, sendo um de representação estadual (13) e outro em nível nacional, realizado somente com adultos (14). Assim, a presente pesquisa teve como objetivo analisar a tendência temporal dos indicadores epidemiológicos e os fatores associados ao abandono de tratamento e óbito pela tuberculose, em pessoas em situação de rua.

## Métodos

### Delineamento e contexto

Trata-se de um estudo transversal com dados sobre a tuberculose no Brasil e macrorregiões do período de janeiro de 2014 a dezembro de 2022. No ano de 2014, houve a inserção de novas variáveis dentro do Sistema de Informação de Agravos de Notificação (Sinan), entre elas, pessoas em situação de rua.

### Participantes

Foram considerados os casos confirmados de tuberculose em pessoas em situação de rua, segundo dados da ficha de notificação da tuberculose. 

A fim de extrair os casos de tuberculose em pessoas em situação de rua, foram levantados todos os casos da doença e selecionados aqueles classificados como “sim” na variável “pessoa em situação de rua”. Assim, foram excluídos os seguintes casos, por não condizerem com os objetivos da pesquisa: (i) pessoas que não eram de situação de rua; (ii) casos em que não havia informação na variável situação de encerramento ou que foram classificados como “mudança de esquema”, “falência”, “mudança de diagnóstico”, “tuberculose drogarresistente” e “transferência”. Além disso, foram excluídos dois casos por classificação incorreta da Classificação Internacional de Doenças – 10ª revisão (CID-10), a saber, A24 e A31, e dois casos duplicados (Figura Suplementar 1).

### Variáveis

A variável dependente foi abandono de tratamento, resultante do agrupamento de abandono de tratamento e abandono primário

Foram incluídas as seguintes variáveis faixa etária (em anos: <19, 20-39, 40-59, ≥60), sexo (masculino, feminino, ignorado), região do país (Norte, Nordeste, Sudeste, Sul e Centro-Oeste), ano de notificação (2014-2022), raça/cor da pele (branca, preta, parda, amarela e indígena, ignorado), escolaridade (analfabeto, ensino fundamental incompleto, ensino fundamental completo, ensino médio incompleto, ensino médio completo, ensino superior incompleto e ensino superior completo, ignorado); clínicas – tipo de entrada (caso novo, retratamento), forma clínica (pulmonar, extrapulmonar, mista, ignorado), aids (sim, não, ignorado), diabetes (sim, não, ignorado), drogas ilícitas (sim, não, ignorado), tabagismo (sim, não, ignorado), tratamento diretamente observado(sim, não, ignorado), baciloscopia de escarro (positiva, negativa, não realizada, inconclusiva, não se aplica), cultura de escarro (positiva, negativa, não realizada, inconclusiva, não se aplica), sorologia anti-HIV (positiva, negativa, não realizada, inconclusiva, não se aplica), teste rápido molecular (positivo para tuberculose sensível a rifampicina; resistente à rifampicina; não realizado; negativo, ignorado), encerramento do caso (cura, abandono de tratamento, abandono primário e óbito por tuberculose).

Adicionalmente, foi calculado um sistema de classificação empírico para avaliar a qualidade do acompanhamento dos casos seguindo as recomendações da Sociedade Brasileira de Pneumologia e Tisiologia (15). O indicador combina cinco recomendações: (1) se o caso notificado realizou baciloscopia de controle no 2º mês de tratamento; (2) baciloscopia de controle no 4º mês de tratamento; (3) baciloscopia de controle no 6º mês de tratamento; (4) se houve registro de exames de contatos; e (5) se houve tratamento diretamente observado. A classificação é dividida em quatro, a saber: acompanhamento insuficiente, regular, bom e excelente. A variável dependente foi o encerramento do caso (cura, abandono de tratamento e óbito por tuberculose).

Nos dados agregados, foram calculados os seguintes indicadores epidemiológicos, mediante padrão do Ministério da Saúde (2): taxa de incidência e mortalidade, proporção de cura e abandono de tratamento. As taxas de incidência e mortalidade seguiram a fórmula:


$$\frac{o_{i}}{p_{i}}\times \text{100 mil}$$

Onde: o_i_ – casos novos ou óbitos por tuberculose em pessoas em situação de rua em determinado local e período; e p_i_ – população em situação de rua no mesmo local e período. Somente para esse cálculo, foram considerados apenas os casos classificados como caso novo, pós-óbito e não sabe, na variável “tipo de entrada”. Além disso, os óbitos não foram extraídos do Sistema de Informação sobre Mortalidade (SIM), devido à impossibilidade de classificar as mortes segundo população de rua. 

As proporções de cura e abandono seguiram a fórmula:


$$\frac{o_{i}}{t_{i}}\times 100$$

Onde: o_i_ – ocorrências brutas dos casos totais que evoluíram para cura e abandono de tratamento em pessoas em situação de rua, em um local e período; e t_i_ – total de casos de tuberculose em pessoas em situação de rua no mesmo local e período.

### Fonte de dados e mensuração

Os dados referentes aos casos confirmados de tuberculose foram extraídos do Sinan, enquanto a estimativa da população em situação de rua foi proveniente do Instituto de Pesquisa Econômica Aplicada, estimados por meio de coleta de dados oficiais de prefeituras, número de pessoas em situação de rua cadastradas no Cadastro Único e projeções baseadas em dados anteriores. Essas informações estão disponíveis no período de 2012 a 2022, segundo Brasil, macrorregiões e porte dos municípios (pequeno I até metrópole) (16).

Todos os dados foram acessados em setembro de 2023, da seguinte forma (Figura suplementar 1):

- Acesso aos bancos anuais (2014 a 2022) das fichas de notificação de tuberculose no site do Departamento de Informática do Sistema Único de Saúde – dados em formato .dbc;
- Importação dos dados baixados para conversão para .dbf, utilizando-se o TabWin;
- União dos bancos de dados anuais (2014-2022) por meio do TabWin, na seção “Ver .dbf” -> “Adicionar registros”;
- Tabulação dos dados segundo ano e características sociodemográficas (dados agregados);
- Exportação dos microdados para tratamento em planilha do Microsoft Excel.

Nas variáveis sorologia anti-HIV, cultura de escarro e baciloscopia, as classificações inconclusivas e não se aplica, além das ignoradas nas demais variáveis, foram transformadas em dados sem informações.

### Viés

Considerando-se a presença de dados faltantes no estudo e visando à melhoria do modelo preditivo, foi realizado procedimento de imputação múltipla, partindo do pressuposto de que as perdas são do tipo ao acaso, fato comum em estudos dessa natureza (17). Por meio da inserção de todas as variáveis disponíveis e usando o método de correspondência preditiva, foram propostos dez modelos de imputação, para maior poder e precisão (18).

### Análise dos dados

A tendência temporal foi estimada pela de regressão de Prais-Winsten. Todos os indicadores epidemiológicos foram transformados em escala logarítmica de base 10. Com base nos resultados, estimou-se a variação percentual anual (VPA) e o intervalo de confiança de 95% (IC95%) (19).

A variação percentual foi interpretada da seguinte forma (19):

- Tendência crescente: variação positiva e modelo estatisticamente significativo (p-valor<0,05);
- Tendência decrescente: variação negativa e modelo estatisticamente significativo (p-valor<0,05);
- Tendência estacionária: modelo não significativo (p-valor>0,05).

Nesta pesquisa, os valores de Durbin-Watson, após a correção da autocorrelação, foram reportados para assegurar as interpretações da tendência, considerando-se como confiáveis valores entre 1,5 e 2,5. A tendência foi calculada no Stata versão 17.

A estimativa dos fatores associados foi realizada pela regressão logística multinominal, utilizando-se o método de entrada enter. Considerou-se como variável dependente a situação de encerramento dos casos classificados como cura, abandono de tratamento e óbito por tuberculose.

A categoria “cura” foi utilizada como referência, sendo comparada com as categorias de abandono de tratamento e óbito por tuberculose. 

Inicialmente, foi proposto um modelo de regressão logística com todas as variáveis inseridas para calcular a razão de chances (*odds ratio*, OR) e IC95%, e progressivamente foram testados múltiplos modelos, por meio da remoção e reinserção de variáveis, visando ao ajuste do potencial de confusão e interação do modelo. Para comparar os testes, foram analisados o critério de informação bayesiano e o Nagelkerke (*pseudo R*-square). Considerou-se a média do critério bayesiano dos dez bancos imputados como medida comparativa. O modelo ajustado incluiu todos os preditores do modelo final como variáveis de ajuste.

Foram calculadas as razões de chances (*odds ratio*, OR) brutas e ajustadas. As análises foram realizadas no Statistical Package for Social Science versão 25.

## Resultados

Foram inclusas 21.904 pessoas em situação de rua com tuberculose, o que representa 2,7% do total de casos notificados no país (n=820.247). A região Sudeste apresentou a maior proporção de notificações, seguida das regiões Sul, Nordeste, Norte e Centro-Oeste (Tabela 1). Além disso, foram contabilizados um total de 6,3% de dados faltantes (Tabela suplementar 1).

**Tabela 1:** Número de casos de tuberculose em pessoas em situação de rua, segundo variáveis sociodemográficas e clínicas. Brasil, 2014-2022 (n=21.904)

Características	n (%)
**Faixa etária** (anos)	
<19	348 (1,6)
>60	1.258 (5,7)
20-39	10.928 (49,9)
40-59	9.370 (42,8)
Sexo	
Feminino	3.996 (18,2)
Masculino	17.907 (81,8)
Sem informação	1 (0,0)
**Raça/cor da pele**	
Amarela/indígena	233 (1,1)
Branca	5.512 (25,2)
Negra	14.299 (65,3)
Sem informação	1.860 (8,5)
Escolaridade	
Analfabeto	978 (4,5)
Fundamental	9.745 (44,5)
Médio	2.770 (12,6)
Sem informação	8.022 (36,6)
Superior	389 (1,8)
**Região**	
Centro-Oeste	884 (4,0)
Nordeste	3.315 (15,1)
Norte	1.124 (5,1)
Sudeste	12.814 (58,5)
Sul	3.767 (17,2)
**Tipo de entrada**	
Caso novo	8.874 (40,5)
Retratamento	12.736 (58,1)
Sem informação	294 (1,3)
**Forma clínica**	
Extrapulmonar	741 (3,4)
Mista	658 (3,0)
Pulmonar	20.503 (93,6)
Sem informação	2 (0,0)
Aids	
Não	16.154 (73,7)
Sem informação	1.515 (6,9)
Sim	4.235 (19,3)
Alcoolismo	
Não	9.083 (41,5)
Sem informação	1.036 (4,7)
Sim	11.785 (53,8)
Diabetes	
Não	19.699 (89,9)
Sem informação	1.443 (6,6)
Sim	762 (3,5)
**Drogas ilícitas**	
Não	8.188 (37,4)
Sem informação	1.198 (5,5)
Sim	12.518 (57,1)
Tabagismo	
Não	10.676 (48,7)
Sem informação	1.420 (6,5)
Sim	9.808 (44,8)
**Tratamento diretamente observado**	
Não	8.756 (40,0)
Sem informação	5.903 (26,9)
Sim	7.245 (33,1)
**Baciloscopia de escarro**	
Não realizada	3.839 (17,5)
Negativa	5.681 (25,9)
Positiva	11.954 (54,6)
Sem informação	430 (2,0)
**Cultura de escarro**	
Não realizada	2.174 (9,9)
Negativa	10.681 (48,8)
Positiva	8.456 (38,6)
Sem informação	593 (2,7)
**Sorologia anti**-HIV	
Não realizada	13.863 (63,3)
Negativa	3.243 (14,8)
Positiva	4.543 (20,7)
Sem informação	255 (1,2)
Acompanhamento	
Bom	244 (1,1)
Excelente	32 (0,1)
Insuficiente	18.955 (86,5)
Regular	2.673 (12,2)
**Teste rápido molecular**	
Não realizado	9.799 (44,7)
Negativo	1.111 (5,1)
Positivo para tuberculose sensível à rifampicina	8.606 (39,3)
Resistente à rifampicina	179 (0,8)
Sem informação	2.209 (10,1)
**Encerramento do caso**	
Abandono de tratamento	11.260 (51,4)
Cura	8.653 (39,5)
Óbito pela tuberculose	1.991 (9,1)

No país, a proporção de cura foi de 31,3%; de abandono de tratamento, de 35,2%; a taxa de incidência foi de 1.007,81 casos para cada 100 mil pessoas em situação de rua; e a mortalidade por tuberculose foi de 126,99/100 mil. Na região Sudeste, houve uma redução considerável da taxa de incidência a partir de 2019 (Tabela suplementar 2). Observou-se um padrão visual de redução nas proporções de cura e na incidência, enquanto o abandono de tratamento e a mortalidade aparentam estagnação (Figura 1).

**Figura 1:** Proporções de cura (A) e abandono de tratamento (B), taxas de incidência (C) e de mortalidade (D) por 100 mil habitantes da tuberculose em pessoas em situação de rua, por macrorregião do país. Brasil, 2014-2022 (n=21.904)

A maioria da população de rua estudada foi do sexo masculino (81,8%), adultos jovens (49,9%), negros (65,3%), com ensino fundamental (44,5%) e residentes no Sudeste (58,5%). As características clínicas de predominância foram: casos em retratamento (58,1%), de forma clínica pulmonar (93,6%), alcoolismo (53,8%), tabagismo (48,7%) e drogas ilícitas presentes (57,1%), tratamento diretamente observado ausente (40,0%), baciloscopia positiva (54,6%), cultura de escarro negativa (48,8%), sorologia anti-HIV não realizadas (63,3%), e acompanhamento insuficiente (86,5%) (Tabela 1).

As proporções de cura apresentaram tendência de redução em todo o território nacional (VPA -5,8; IC95% -7,9; -3,8), com destaque para as macrorregiões Nordeste (VPA -8,5; IC95% -11,0; -6,0) e Norte (VPA -7,7; IC95% -12,2; -2,8). O indicador de abandono apresentou tendência estacionária, enquanto na região Sudeste a incidência (VPA -6,2; IC95% -8,9; -3,5) e a mortalidade diminuíram (VPA -5,3; IC95% -10,0; -0,4). Não houve evidências de vieses de autocorrelação nesses modelos (Durbin-Watson >1,5) (Tabela 2). 

**Tabela 2:** Variação percentual anual (VPA) e intervalos de confiança (IC95%) da proporção de cura e abandono de tratamento, taxa de incidência e mortalidade (por 100 mil) por tuberculose em pessoas em situação de rua. Brasil, 2014-2022 (n=21.904)

Indicadores	VPA (IC95%)	p-valor	Durbin-Watson	Interpretação
**Proporção de cura**				
Norte	-7,7 (-12,2; -2,8)	0,008	1,465	Decrescente
Nordeste	-8,5 (-11,0; -6,0)	<0,001	1,762	Decrescente
Sudeste	-4,0 (-5,9; -1,9)	0,003	1,403	Decrescente
Sul	-6,8 (-8,2; -5,3)	<0,001	1,801	Decrescente
Centro-Oeste	-6,9 (-9,5; -4,3)	<0,001	2,020	Decrescente
Brasil	-5,8 (-7,9; -3,8)	<0,001	1,421	Decrescente
**Proporção de abandono**				
Norte	2,4 (-2,9; 7,9)	0,323	1,525	Estacionária
Nordeste	0,1 (-0,4; 0,4)	0,818	1,765	Estacionária
Sudeste	0,4 (-0,7; 1,6)	0,388	1,921	Estacionária
Sul	-3,0 (-6,5; 0,6)	0,087	1,382	Estacionária
Centro-Oeste	-1,1 (-2,9; 0,8)	0,209	1,804	Estacionária
Brasil	-0,2 (-1,1; 0,7)	0,623	1,351	Estacionária
**Taxa de incidência**				
Norte	-3,9 (-16,6; 10,9)	0,535	1,379	Estacionária
Nordeste	4,9 (-8,9; 20,9)	0,449	1,523	Estacionária
Sudeste	-6,2 (-8,9; -3,5)	<0,001	1,554	Decrescente
Sul	-2,0 (-7,9; 4,7)	0,454	1,504	Estacionária
Centro-Oeste	6,4 (-3,3; 17,1)	0,169	1,273	Estacionária
Brasil	-3,5 (-8,2; 1,3)	0,128	1,369	Estacionária
**Taxa de mortalidade**				
Norte	-6,2 (-20,7; 11,1)	0,401	1,466	Estacionária
Nordeste	12,6 (-0,3; 27,1)	0,053	1,435	Estacionária
Sudeste	-5,3 (-10,0; -0,4)	0,038	1,816	Decrescente
Sul	7,2 (-1,4; 16,5)	0,091	1,645	Estacionária
Centro-Oeste	19,7 (2,5; 40,0)	0,029	1,397	Crescente

Quatro modelos de regressão logística multinominal foram propostos, tendo sido removidas as variáveis indicador de acompanhamento, aids e macrorregião (Tabela suplementar 3). O modelo final foi estatisticamente significativo (p-valor<0,001) (Tabelas 3 e 4).

Maiores chances de abandono de tratamento em pessoas em situação de rua foram observadas em indivíduos de 20-39 anos (OR 0,69; IC95% 1,31; 2,17), que faziam uso de drogas ilícitas (OR 1,57; IC95% 1,46; 1,69) e sem tratamento diretamente observado (OR 3,93; IC95: 3,67; 4,20) (Tabela 3). Foram identificadas maiores chances de óbito entre pessoas acima de 60 anos (OR 3,10 IC95% 1,96; 4,90), infectadas com forma clínica mista (OR 2,66; IC95% 2,05; 3,46), que fazem uso de álcool (OR 1,34; IC95% 1,18; 1,51), que usam drogas ilícitas (OR 1,18; IC95% 1,05; 1,34) e sem tratamento diretamente observado (OR 3,46; IC95% 2,98; 4,02) (Tabela 4).

**Tabela 4:** Razão de chances (*odds ratio*, OR) e intervalos de confiança de 95% (IC95%) brutas e ajustadas do óbito por tuberculose, pelas variáveis sociodemográficas e clínicas, em pessoas em situação de rua. Brasil, 2014-2022 (n=21.904)

Variável	OR bruta (IC95%)	p-valor	OR ajustada (IC95%)	p-valor
**Faixa etária** (anos)				
<19	1,00		1,00	
>60	2,91 (1,89; 4,46)	<0,001	3,10 (1,96; 4,90)	<0,001
20-39	1,15 (0,76; 1,74)	0,498	1,29 (0,84; 2,00)	0,249
40-59	1,65 (1,09; 2,48)	0,017	1,82 (1,18; 2,81)	0,007
Sexo				
Feminino	1,00		1,00	
Masculino	1,37 (1,19; 1,58)	<0,001	1,25 (1,07; 1,46)	0,005
**Raça/cor da pele**				
Amarela/indígena	0,89 (0,56; 1,42)	0,636	0,79 (0,48; 1,29)	0,34
Branca	1,00		1,00	
Negra	1,06 (0,94; 1,19)	0,336	1,03 (0,91; 1,16)	0,696
**Escolaridade**				
Analfabeto	4,59 (2,36; 8,92)	<0,001	4,13 (2,11; 8,08)	<0,001
Fundamental	2,99 (1,65; 5,43)	<0,001	2,88 (1,62; 5,13)	<0,001
Médio	2,38 (1,32; 4,29)	0,006	2,41 (1,37; 4,24)	0,004
Superior	1,00		1,00	
**Tipo de entrada**				
Caso novo	1,00		1,00	
Retratamento	0,88 (0,79; 0,98)	0,019	0,78 (0,69; 0,87)	<0,001
**Forma clínica**				
Extrapulmonar	1,05 (0,82; 1,35)	0,688	0,96 (0,73; 1,28)	0,793
Mista	2,14 (1,90; 2,42)	<0,001	2,66 (2,05; 3,46)	<0,001
Pulmonar	1,00		1,00	
Alcoolismo				
Não	1,00		1,00	
Sim	1,27 (1,14; 1,41)	<0,001	1,34 (1,18; 1,51)	<0,001
Diabetes				
Não	1,00		1,00	
Sim	1,28 (1,01; 1,62)	0,044	1,07 (0,83; 1,37)	0,611
**Drogas ilícitas**				
Não	1,00		1,00	
Sim	0,89 (0,81; 0,99)	0,029	1,18 (1,05; 1,34)	0,008
Tabagismo				
Não	1,00		1,00	
Sim	0,97 (0,88; 1,08)	0,583	0,91 (0,81; 1,03)	0,139
**Tratamento diretamente observado**				
Não	3,46 (2,95; 4,06)	<0,001	3,46 (2,98; 4,02)	<0,001
Sim	1,00		1,00	
**Baciloscopia de escarro**				
Não realizada	0,44 (0,37; 0,51)	<0,001	0,52 (0,44; 0,62)	<0,001
Negativa	1,00		1,00	
Positiva	0,63 (0,57; 0,70)	<0,001	0,70 (0,62; 0,79)	<0,001
**Cultura de escarro**				
Não realizada	0,27 (0,22; 0,33)	<0,001	0,35 (0,28; 0,44)	<0,001
Negativa	1,00		1,00	
Positiva	0,38 (0,34; 0,42)	<0,001	0,46 (0,41; 0,52)	<0,001
**Sorologia anti**-HIV				
Não realizada	0,25 (0,22; 0,29)	<0,001	0,32 (0,28; 0,37)	<0,001
Negativa	1,00		1,00	
Positiva	0,15 (0,12; 0,18)	<0,001	0,16 (0,13; 0,19)	<0,001
**Teste rápido molecular**				
Não realizado	2,66 (1,94; 3,63)	<0,001	1,75 (1,26; 2,43)	<0,001
Negativo	1,00		1,00	
Resistente à rifampicina	1,83 (0,96; 3,52)	0,069	1,43 (0,71; 2,87)	0,318
Sensível à rifampicina	1,60 (1,18; 2,18)	0,003	1,44 (1,05; 1,99)	0,025

**Tabela 3:** Razão de chances (odds ratio, OR) e intervalos de confiança de 95% (IC95%) brutas e ajustadas do abandono de tratamento da tuberculose, pelas variáveis sociodemográficas e clínicas, em pessoas em situação de rua. Brasil, 2014-2022 (n=21.904)

Variável	OR bruta (IC95%)	p-valor	OR ajustada (IC95%)	p-valor
**Faixa etária** (anos)				
<19	1,00		1,00	
>60	0,69 (0,54; 0,89)	0,005	0,79 (0,59; 1,05)	0,107
20-39	2,10 (1,68; 2,62)	<0,001	1,69 (1,31; 2,17)	<0,001
40-59	1,30 (1,04; 1,63)	0,022	1,18 (0,92; 1,53)	0,191
Sexo				
Feminino	1,00		1,00	
Masculino	0,76 (0,71; 0,82)	<0,001	1,00 (0,92; 1,09)	0,966
**Raça/cor da pele**				
Amarela/indígena	0,68 (0,51; 0,90)	0,007	0,71 (0,51; 1,00)	0,051
Branca	1,00		1,00	
Negra	1,11 (1,04; 1,19)	0,002	1,07 (0,99; 1,16)	0,089
Escolaridade				
Analfabeto	1,25 (0,96; 1,62)	0,099	1,43 (1,08; 1,88)	0,015
Fundamental	1,29 (1,02; 1,64)	0,038	1,21 (0,93; 1,56)	0,156
Médio	1,13 (0,87; 1,46)	0,361	1,01 (0,77; 1,31)	0,958
Superior	1,00		1,00	
**Tipo de entrada**				
Caso novo	1,00		1,00	
Retratamento	0,51 (0,48; 0,54)	<0,001	0,55 (0,51; 0,58)	<0,001
**Forma clínica**				
Extrapulmonar	0,74 (0,63; 0,86)	<0,001	0,65 (0,54; 0,77)	<0,001
Mista	1,09 (0,92; 1,30)	0,312	0,86 (0,71; 1,05)	0,138
Pulmonar	1,00		1,00	
Alcoolismo				
Não	1,00		1,00	
Sim	1,04 (0,98; 1,10)	0,23	0,95 (0,88; 1,02)	0,166
Diabetes				
Não	1,00		1,00	
Sim	0,74 (0,63; 0,86)	<0,001	0,87 (0,74; 1,03)	0,109
**Drogas ilícitas**				
Não	1,00		1,00	
Sim	1,83 (1,73; 1,95)	<0,001	1,57 (1,46; 1,69)	<0,001
Tabagismo				
Não	1,00		1,00	
Sim	1,20 (1,13; 1,27)	<0,001	1,11 (1,03; 1,20)	0,009
Tratamento diretamente observado				
Não	4,05 (3,80; 4,32)	<0,001	3,93 (3,67; 4,20)	<0,001
Sim	1,00		1,00	
**Baciloscopia de escarro**				
Não realizada	0,84 (0,77; 0,91)	<0,001	0,93 (0,84; 1,02)	0,135
Negativa	1,00		1,00	
Positiva	0,83 (0,77; 0,88)	<0,001	0,90 (0,83; 0,98)	0,01
**Cultura de escarro**				
Não realizada	0,58 (0,53; 0,64)	<0,001	0,55 (0,49; 0,62)	<0,001
Negativa	1,00		1,00	
Positiva	0,69 (0,65; 0,73)	<0,001	0,68 (0,63; 0,73)	<0,001
**Sorologia anti**-HIV				
Não realizada	0,40 (0,36; 0,44)	<0,001	0,42 (0,38; 0,47)	<0,001
Negativa	1,00		1,00	
Positiva	0,79 (0,71; 0,88)	<0,001	0,64 (0,57; 0,72)	<0,001
**Teste rápido molecular**				
Não realizado	0,98 (0,86; 1,11)	0,727	0,88 (0,76; 1,01)	0,074
Negativo	1,00		1,00	
Resistente à rifampicina	1,12 (0,80; 1,58)	0,501	0,98 (0,67; 1,43)	0,902
Sensível à rifampicina	1,02 (0,90; 1,16)	0,701	1,11 (0,96; 1,28)	0,173

## Discussão

Nesta pesquisa, foi identificado um cenário preocupante e desfavorável da tuberculose em pessoas em situação de rua, com indicadores epidemiológicos longe das metas estabelecidas pela OMS, de no mínimo 85% de cura, máximo de 5% de abandono e 1,00 morte por 100 mil habitantes (11). Foi identificada redução anual nas proporções de cura em todo o território brasileiro, e redução de incidência e mortalidade somente na região Sudeste. Fatores que mais elevam a chance de abandono são a ausência de tratamento diretamente observado, faixa etária de 20-39 anos e uso de drogas ilícitas, enquanto as chances de óbito são maiores entre pessoas acima de 60 anos, analfabetos, com forma clínica mista e com tratamento diretamente observado ausente.

A principal limitação deste estudo refere-se ao uso de dados secundários, pela subnotificação e incompletude de dados, especialmente nessa população, que pode ser de difícil captação e acompanhamento. Os dados referentes aos óbitos seriam mais confiáveis se obtidos pelo sistema de informação sobre mortalidade. Nesse sistema não é possível identificar as pessoas em situação de rua. Outra limitação refere-se à possível menor qualidade dos dados no ano de 2014, quando houve a inserção das novas variáveis, além dos anos de 2020-2022, pelo aumento da subnotificação devido à pandemia da covid-19. Potencialmente algumas duplicações não puderam ser excluídas devido à natureza dos dados que não apresentam informações pessoais. 

As taxas de incidência e mortalidade, a proporção de cura e abandono de tratamento em pessoas em situação de rua identificados na presente pesquisa são desfavoráveis e discrepantes, em comparação a outros grupos vulneráveis. No Brasil, pessoas privadas de liberdade, no período de 2013-2015, apresentaram incidência de 540,90/100 mil (20), enquanto em uma pesquisa em indígenas (2011-2017) a incidência média foi de 109,00/100 mil (21). A incidência também foi superior à identificada nos Estados Unidos (2011-2016), de 36,00/100 mil (22). Uma possível justificativa para essa discrepância é a combinação entre subnotificação e maior vulnerabilidade social no Brasil. Fatores como maior densidade populacional em áreas urbanas, políticas públicas menos efetivas e barreiras no acesso aos serviços de saúde podem contribuir para uma incidência mais elevada. A tuberculose nessa população é complexa, quando comparada ao restante da população, pela existência de situações precárias de segurança, descanso e alimentação inadequados, presença de HIV/aids, hipertensão arterial e uso de drogas, que são recorrentes nesse grupo e contribuem para dificuldades no tratamento, elevando as chances de desfechos desfavoráveis (9). Essa realidade é agravada pela marginalização, acolhimento inadequado, distanciamento das políticas públicas e o acesso à saúde dificultado, fato que compromete a assistência e acarreta contextos negativos.

Na presente pesquisa, a proporção de cura apresentou redução nos últimos nove anos no país e macrorregiões. A literatura carece de evidências sobre a tendência da cura em pessoas em situação de rua; porém, em pessoas vivendo com HIV, o padrão de tendência foi semelhante, com tendência decrescente de 1,1% (IC95% -1,4; -0,8) ao ano entre 2002-2021 no Brasil (23). A redução na taxa de cura é preocupante e sugere a necessidade de intervenções imediatas pelas autoridades de saúde pública. Esse cenário pode refletir dificuldades contínuas na melhoria da adesão ao tratamento, especialmente em pessoas em situação de rua, as quais são agravadas por questões sociais e econômicas que impactam diretamente a capacidade dos indivíduos de seguir regimes terapêuticos rigorosos. Dessa forma, o tratamento da doença nessa população demanda cuidado e apoio mais intensos dos profissionais e das instituições de saúde. A falta de adesão é um sério problema, entendido como multicausal (24); portanto, o fortalecimento das estratégias de controle da tuberculose deve ser focado na prevenção, rastreamento, busca ativa de casos e melhoria do acompanhamento até a cura.

As taxas de incidência e mortalidade apresentaram redução anual no Sudeste. Resultados semelhantes foram encontrados em um estudo sobre incidência nos estados do Sudeste entre 2001-2017 (4) e sobre mortalidade no período de 2005-2019 (5). Isso pode ser justificado pela maior efetividade de políticas públicas da região, além do fortalecimento dos programas de controle da tuberculose, aliado a investimentos em infraestrutura de saúde e maior cobertura da atenção primária. Ainda assim, a região Sudeste apresentou maiores concentrações de pessoas em situação de rua que são expostas a precárias condições de vida e comorbidades (25). A política de proteção social à pessoa com tuberculose mostra-se relevante ao fortalecer o suporte à pessoa em situação de rua por meio da inclusão social, da garantia de acesso aos direitos sociais e humanos e do enfrentamento da pobreza (26). A Política Nacional para a População em Situação de Rua, por seu turno, visa promover o respeito à dignidade humana, a integração das políticas públicas em todos os níveis de governo, e o acesso amplo e seguro a serviços de saúde, assistência social e programas de transferência de renda (27). Apesar desse cenário, os desafios para o controle da tuberculose nessa população ainda são diversos, como recursos humanos diminuídos, despreparo profissional, e fragmentação do cuidado. 

A ausência do tratamento diretamente observado foi preditor para o aumento das chances de abandono de tratamento e óbito pela tuberculose, e foi oferecido para menos da metade da população. Resultado semelhante foi observado nos Estados Unidos entre 2014-2016, onde as chances de completar o tratamento da tuberculose latente com tratamento diretamente observado foi 40% maior quando comparado à autoadministração em pessoas em situação de rua (28). A OMS destaca essa estratégia como importante para o aumento nos desfechos favoráveis (29). É papel municipal e local a identificação de melhores estratégias para a abordagem desse grupo, sendo a intersetorialidade requisito básico para melhores indicadores. O mapeamento das redes de atendimento, a busca de parcerias para detecção, diagnóstico e tratamento, a definição das unidades de referência, a busca ativa e a capacitação dos profissionais envolvidos são relevantes para mitigar desfechos negativos do agravo (30). Auxílios para diminuição do impacto econômico do tratamento ou a garantia de transporte e alimentação fazem-se importante para garantir atenção abrangente e de longo prazo.

Neste estudo foi observado que mais da metade da população fez uso de álcool, tabaco e drogas ilícitas, e esses fatores foram relacionados ao aumento dos desfechos desfavoráveis da doença. Esses resultados corroboram um estudo realizado em São Paulo (13). O uso de tais substâncias contribui para a elevação da vulnerabilidade dessa população quanto à tuberculose e, por consequência, o abuso delas é potencializado por determinantes sociais, como o modo de viver nas ruas, a luta pela sobrevivência, condições precárias de sono, pobreza e estigma (12). Apesar de o sistema de saúde brasileiro ser público, deve-se considerar que essa população prioriza a busca por condições básicas de vida, como abrigo e comida (9), o que pode atrasar e dificultar a busca por assistência. Tornam-se relevantes estratégias como programas sociais para o auxílio aos dependentes integrados aos serviços de saúde, por meio de campanhas educativas, redução de danos, reabilitação e reintegração social.

A forma clínica mista aumentou a chance de óbito em duas vezes, comparada à forma pulmonar. Dados semelhantes foram encontrados em um estudo brasileiro (n=2.096) realizado com população indígena jovem no período de 2006-2016 (OR 5,60; IC95% 2,80; 11,40) (31). A forma extrapulmonar é particularmente mais difícil de ser identificada, pela baixa carga bacteriana pulmonar e o desafio na coleta de amostras de escarro (32), além da presença de sinais e sintomas atípicos. Casos de tuberculose mista merecem atenção, tendo em vista que esta é frequente entre pessoas vivendo com HIV/aids e com comprometimento imunológico (33). Considerando a complexidade de se rastrear a forma extrapulmonar, sugere-se aprimoramentos nas redes de atenção para busca ativa e tratamento individualizado e adequado às necessidades das pessoas em situação de rua, visando reduzir as chances de morte. 

As proporções de cura da tuberculose apresentaram redução em todo o território nacional, com destaque para as macrorregiões Nordeste e Norte, no período de 2014 a 2022. O indicador de abandono não apresentou variação anual, enquanto a incidência e a mortalidade apresentaram redução somente no Sudeste. Além disso, houve tendência crescente anual da mortalidade no Centro-Oeste. Diversos fatores contribuíram para um maior risco de abandono de tratamento e óbito por tuberculose, a saber: faixa etária, analfabetismo, retratamento dos casos, uso de drogas ilícitas, tabagismo, alcoolismo e ausência de tratamento diretamente observado.
